# Quantitative X-ray phase contrast waveguide imaging of bacterial endospores^1^

**DOI:** 10.1107/S1600576715003593

**Published:** 2015-03-24

**Authors:** R. N. Wilke, M. Hoppert, M. Krenkel, M. Bartels, T. Salditt

**Affiliations:** a University of Göttingen, Institute for X-ray Physics, Friedrich-Hund-Platz 1, 37077 Göttingen, Germany; b University of Göttingen, Institute of Microbiology and Genetics, Grisebachstrasse 8, 37077 Göttingen, Germany; c Philips Research, Hamburg, Germany

**Keywords:** phase retrieval, waveguide imaging, contrast transfer function, holotomography, *Bacillus thuringiensis*, *Bacillus subtilis*, endospores

## Abstract

Quantitative waveguide-based X-ray phase contrast imaging has been carried out on the level of single, unstained, unsliced and freeze-dried bacterial cells of *Bacillus thuringiensis* and *Bacillus subtilis* using hard X-rays of 7.9 keV photon energy. The cells have been prepared in the metabolically dormant state of an endospore. The quantitative phase maps obtained by iterative phase retrieval using a modified hybrid input–output algorithm allow for mass and mass density determinations on the level of single individual endospores but include also large field of view investigations. Additionally, a direct reconstruction based on the contrast transfer function is investigated, and the two approaches are compared. Depending on the field of view and method, a resolution down to 65 nm was achieved at a maximum applied dose of below 5 × 10^5^ Gy. Masses in the range of about ∼110–190 (20) fg for isolated endospores have been obtained.

## Abstract 2

Quantitative X-ray phase contrast imaging uniquely offers quantitative imaging information in terms of electron density maps allowing for mass and mass density determinations of soft biological samples (‘weighing with light’). Here, it was carried out using coherent X-ray waveguide illumination, yielding values of the mass and mass density of freeze-dried bacterial endospores (*Bacillus* spp.).

## 1. Introduction   

Coherent X-ray diffraction imaging (CDI) has reached a resolution level where internal features of single eukaryotic and prokaryotic cells can be analysed, making it a powerful tool for quantitative microscopic investigations on the scale of a few nanometres. In particular, recent results in the energy region of soft X-rays (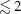
 keV) promise imaging at the limit of the wavelength on radiation hard samples (Barty *et al.*, 2008 ▶; Chapman *et al.*, 2006 ▶; Maiden *et al.*, 2013 ▶; Miao *et al.*, 1999 ▶; Shapiro *et al.*, 2014 ▶), as well as imaging of soft biological samples at slightly lower but still fairly high resolution (Huang *et al.*, 2009 ▶; Miao *et al.*, 2003 ▶; Shapiro *et al.*, 2005 ▶; Nelson *et al.*, 2010 ▶). The energy range of medium and hard X-rays also facilitates probing of comparably thick specimens under ambient conditions. This regime suffers more strongly from the unavailability of efficient imaging lenses and thus relies on alternative imaging schemes such as CDI. Similarly to the progress using soft X-rays, there are promising results on radiation hard samples (Dierolf *et al.*, 2010 ▶; Guizar-Sicairos *et al.*, 2014 ▶; Holler *et al.*, 2014 ▶; Rodenburg *et al.*, 2007 ▶; Schropp *et al.*, 2012 ▶; Suzuki *et al.*, 2014 ▶; Takahashi *et al.*, 2013 ▶; Thibault *et al.*, 2008 ▶; Vila-Comamala *et al.*, 2011 ▶; Wilke *et al.*, 2013 ▶, 2014 ▶) and radiation-sensitive biological samples (Bartels *et al.*, 2012 ▶; Jiang *et al.*, 2010 ▶; Jones *et al.*, 2013 ▶; Lima *et al.*, 2013 ▶; Nam *et al.*, 2013 ▶; Song *et al.*, 2008 ▶; Wilke *et al.*, 2012 ▶). However, the experiments can still be optimized in terms of resolution and, especially, dose. An important alternative to phase retrieval from far-field diffraction patterns can be reached by switching to the Fresnel and holographic regime, where algorithms tend to converge very robustly (Giewekemeyer *et al.*, 2011 ▶; Gureyev, 2003 ▶; Williams *et al.*, 2006 ▶, 2007 ▶). Moreover, imaging appears to be surprisingly dose efficient (Bartels *et al.*, 2012 ▶, 2015 ▶; Jones *et al.*, 2013 ▶, 2014 ▶; Putkunz *et al.*, 2011 ▶).

Consequently, the phase retrieval experiments within this work are carried out in the holographic regime by using an X-ray waveguide (Di Fonzo *et al.*, 1998 ▶; Lagomarsino *et al.*, 1997 ▶; Müller *et al.*, 2000 ▶; Pfeiffer *et al.*, 2002 ▶), which is an optical element for preparing a quasi-point-source with a size well below 100 nm in the hard X-ray regime (Jarre *et al.*, 2005 ▶; Krüger *et al.*, 2012 ▶; Ollinger *et al.*, 2007 ▶). Owing to the filtering of the coupled X-rays by propagation through the waveguide (De Caro *et al.*, 2003 ▶; Fuhse *et al.*, 2004 ▶; Osterhoff & Salditt, 2011 ▶; Tsanaktsidis *et al.*, 2013 ▶), the prepared quasi-point-source is ideally suited for imaging in the holographic regime in two and three dimensions (Bartels *et al.*, 2012 ▶, 2015 ▶; Bartels, 2013 ▶; Fuhse *et al.*, 2006 ▶; Krenkel *et al.*, 2013 ▶; Krüger *et al.*, 2012 ▶). The smooth illumination function of the waveguide (Fuhse, 2006 ▶; Krüger, 2011 ▶; Neubauer *et al.*, 2014 ▶) is an essential ingredient for the phase retrieval methods that rely on normalizing the diffraction data by the empty beam image in order to obtain, for example, a normalized hologram. In contrast to techniques like ptychography (Guizar-Sicairos & Fienup, 2008 ▶; Robisch & Salditt, 2013 ▶; Rodenburg *et al.*, 2007 ▶; Stockmar *et al.*, 2013 ▶; Thibault *et al.*, 2008 ▶) that make use of translational diversity in the probe, waveguide holographic imaging benefits from a very smooth illumination function that allows for eliminating the diffraction signal from the illuminating wavefield prior to the iterative reconstruction step of the holographic data. In this way, the convolution effects from the illumination function become negligible (Hagemann *et al.*, 2014 ▶; Homann *et al.*, 2015 ▶). In addition to iterative phase retrieval methods, the imaging regime allows one to use non-iterative direct phasing methods based on the contrast transfer function (Cloetens *et al.*, 1999 ▶, 2006 ▶; Cloetens, 1999 ▶; Krenkel *et al.*, 2014 ▶; Zabler *et al.*, 2005 ▶) and combined techniques (Gureyev, 2003 ▶; Krenkel *et al.*, 2013 ▶).

In this work the species *Bacillus subtilis* and *Bacillus thuringiensis* are imaged as unsliced, unstained, freeze-dried samples without the use of chemical fixation by quantitative phase retrieval, enabling electron density measurements and mass density determinations. The bacteria of the genus *Bacillus* have become model organisms for microbial life, industrial applications such as biological insecticides and biological warfare (Harwood & Cutting, 1990 ▶; Höfte & Whiteley, 1989 ▶; Meselson *et al.*, 1994 ▶; Spencer, 2003 ▶). They are Gram-positive, rod-shaped, grow under aerobic conditions and are well known for their ability to transform into the metabolically dormant state of an endospore (Errington, 2003 ▶; Harwood & Cutting, 1990 ▶; McKenney *et al.*, 2013 ▶; Nicholson, 2002 ▶; Nicholson *et al.*, 2000 ▶). In the dormant state of an endospore (or simply ‘spore’), the genetic information of the organism is very efficiently stored owing to an increased resistance to, for example, wet and dry heat, desiccation, ionizing radiation such as UV and γ, and chemical agents (Nicholson, 2002 ▶). Reports of longevity include a sample of *B. anthracis* that was originally stored by L. Pasteur in 1888, which showed active growth 68 years later, but even longer resting times are possible (Kennedy *et al.*, 1994 ▶). There are also controversial reports such as ‘revival’ of *Bacillus* spores from insect inclusions of amber that are millions of years old (Cano & Borucki, 1995 ▶).

In contrast to high-resolution studies of endospores using, for example, atomic force microscopy (Plomp *et al.*, 2005*a* ▶, 2007 ▶), X-rays can yield information about the inner structure which can be combined with tomography for a three-dimensional representation. Similarly, transmission electron microscopy (TEM) of biological samples such as spores allows for structural analyses on the nanoscale (Driks, 1999 ▶; Goldman & Tipper, 1978 ▶; Harwood & Cutting, 1990 ▶; Hobot *et al.*, 1985 ▶; McKenney *et al.*, 2013 ▶; Nicholson *et al.*, 2000 ▶). Unfortunately, TEM imaging relies on invasive sample preparation techniques such as staining by heavy metals, chemical fixation, resin embedding and thin sectioning. Cryo-electron microscopy has also advanced to a powerful alternative for high-resolution imaging of biological samples in their near native state (without staining), but the contrast is very weak and this technique does not allow for quantitative analyses (Dubochet, 2012 ▶; Dubochet *et al.*, 1988 ▶; Eltsov & Dubochet, 2005 ▶; Lučić *et al.*, 2005 ▶). Importantly, X-ray phase contrast can thus provide a complementary and quantitative information channel to further elucidate the organization of endospores in a near native state. For instance, the masses of endospores influence the dissemination of spores *via* bioaerosols, this being an important way of spreading infections (Carrera *et al.*, 2008 ▶). At the moment, the determination of mass densities of endospores is typically carried out by density gradient centrifugation methods (Carrera *et al.*, 2008 ▶; Tisa *et al.*, 1982 ▶). One disadvantage of these methods is that they only give access to an average over millions of endospores. However, the ability to study microorganisms on the level of single cells can be of utmost importance when, for example, virulent species are investigated. The scope of this work is to study quantitative X-ray phase contrast measurements on the single-cell level of biologically relevant samples. We investigate advantages and disadvantages of current waveguide-imaging schemes with respect to resolution, dose and quantitativeness, aiming at unprecedented structural analyses of bacterial spores that are not simultaneously accessible by techniques such as atomic force microscopy, transmission electron microscopy and traditional mass density measurement methods. In particular, the weight of single endospores is determined independently of any size models by combining mass density information from the X-ray phase contrast and the imaging information of the contrast. In addition, we propose algorithmic improvements that are aimed at extending the field of view of waveguide holographic imaging by stitching.

The manuscript is organized as follows. First, details about the experimental setup, the theory and the used phase retrieval methods are given. Next, the preparation of the bacterial samples and TEM images, recording of the data and processing steps are described. Before presenting the results, details about dose and mass density determinations are briefly reviewed. The work closes by summarizing the results, comparing the mass measurements with results from other work in different fields and giving an outlook.

## 2. Methods   

### 2.1. Experimental setup   

**Figure 1:**
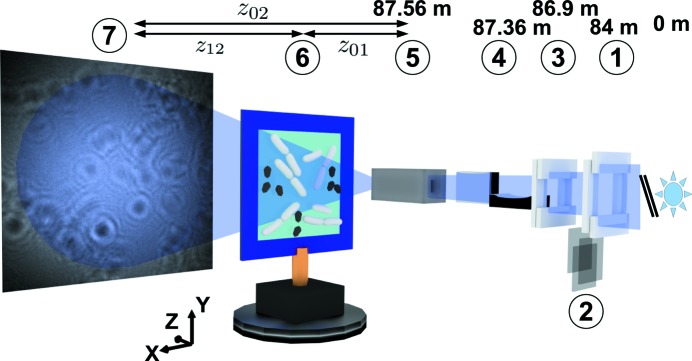
Schematic of the GINIX waveguide setup: downstream of the undulator source and the monochromator (not shown): (1) slits S1, (2) attenuation foils made of Al, (3) slits S2, (4) KB mirror system, (5) X-ray waveguide (entrance in the focal plane of the KB mirrors), (6) bacterial sample at a distance 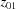 to the waveguide and (7) detection device at a distance 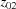 to the waveguide. The flight tube between sample and detector is not shown in the image.

The waveguide-based imaging experiments were carried out during two successive beamtimes with the GINIX (Göttingen Instrument for Nano-Imaging with X-rays) setup at the P10 coherence beamline of the PETRA III synchrotron located at DESY, Hamburg, in Germany (Kalbfleisch *et al.*, 2011 ▶). Monochromatic X-ray undulator radiation with an energy of 7.9 keV was selected by using a double-crystal monochromator [Si(111)]. The beam was focused by a pair of X-ray mirrors in the Kirkpatrick–Baez (KB) geometry (*cf.* Fig. 1 ▶). The beam was then reduced by a waveguide whose guiding channel (air surrounded by silicon) was aligned with respect to the KB focus using a hexapod (SmarAct, Germany). The waveguide was fabricated by e-beam lithography. The channel dimensions are 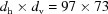
 nm (h denotes horizontal, v denotes vertical) on the entrance side. The channel length is about 1 mm. The source size on the exit of the waveguide was about 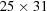
 nm (FWHM, h 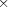 v) (Bartels *et al.*, 2015 ▶). The sample stage follows behind the waveguide. It consisted of a high-precision piezo-electric stage (PI, Germany/SmarAct, Germany) on top of coarse stepper motors (Micos, Germany). The sample is typically positioned at a distance 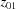, a few millimetres from the waveguide exit. An evacuated flight tube of 5 m length was installed behind the sample stage to minimize scattering effects of X-rays in air. The imaging detector used for collecting the waveguide diffraction data is based on an sCMOS camera (Photonic Science, UK) for visible light. X-ray radiation is detected by visible light from a custom scintillator (15 µm GdOS:Tb, Photonic Science) that is fiber-coupled to the chip. The detector has 1920 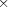 1080 pixels (h 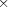 v) with a pixel side length of 6.5 µm. The distances between the waveguide and the detector were either 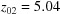
 m or 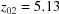
 m, depending on the beamtime. In addition, the single-photon-counting, zero readout noise pixel detector Pilatus 300K (Dectris, Switzerland) was used to determine the photon flux by switching the detectors.

### 2.2. Theory   

**Figure 2:**
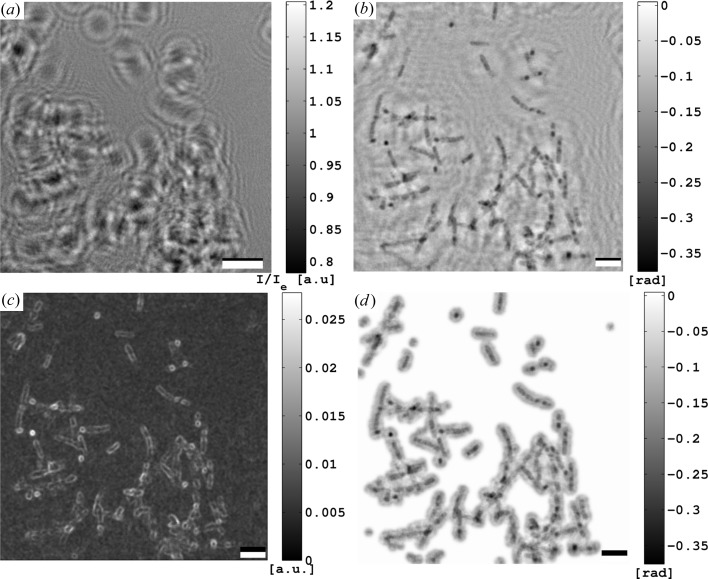
Data of one position on region (A) of the sample with *B. thuringiensis* cell material (*cf.* Table 1): (*a*) hologram 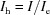 and (*b*) phase of holographic reconstruction which already yields a good impression of the sample structure. For instance, the rod-shaped bacteria (or their remnant empty cell envelopes) can be identified. However, the reconstruction appears distorted owing to the twin image problem. (*c*) The ‘radial’ gradient of the holographic reconstruction in (*b*) used for estimating the support (*cf*. text). (*d*) Product of the final support and holographic phase reconstruction. Scale bars in (*a*) and in (*b*), (*c*), (*d*) denote 1 mm (real detector dimension) and 5 µm (effective geometry), respectively.

The theoretical background for waveguide-based imaging is addressed briefly. The diffraction pattern 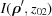 of the object transmission function 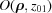 being placed in the divergent beam of a waveguide 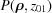 can be described at the detection plane 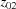 by the Fresnel diffraction integral 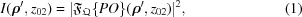
where 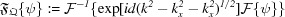 is the angular spectrum approach for free-space propagation of the field ψ of wavenumber *k* by a distance *d*. 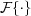 and 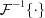 denote the Fourier transform and its inverse, respectively. Owing to the small size of the waveguide exit, the beam can be considered as quasi-point-like (Fuhse, 2006 ▶; Krüger *et al.*, 2012 ▶; Krüger, 2011 ▶). That is, 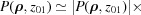 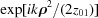. According to the Fresnel scaling theorem (Paganin, 2006 ▶), the diffracted intensity can be described in an effective geometry of coordinates 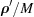 and 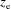 (Bartels, 2013 ▶; Fuhse, 2006 ▶; Krüger, 2011 ▶), 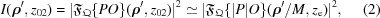
where the geometric magnification *M* of the system and the effective propagation distance 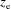 are given by 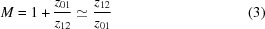
and
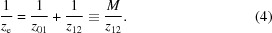
Typically, equation (2) is further simplified by the approximation (Giewekemeyer, 2011 ▶; Giewekemeyer *et al.*, 2011 ▶) 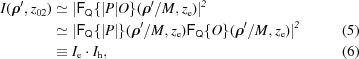
where 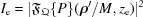 denotes the empty beam intensity and 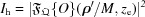 is the hologram of the object. A prerequisite for reconstruction of the waveguide-illuminated Fresnel-regime diffraction data is to record an accurate diffraction pattern of the empty beam. Using equation (6), the holographic data of the object are obtained by dividing the raw data 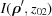 by the empty beam intensity 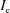 (Bartels *et al.*, 2012 ▶, 2015 ▶; Bartels, 2013 ▶; Giewekemeyer, 2011 ▶; Giewekemeyer *et al.*, 2011 ▶; Hagemann *et al.*, 2014 ▶; Krüger, 2011 ▶): 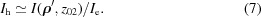
For the present experiment, the empty beam division is exemplified in Fig. 2 ▶(*a*).

### 2.3. Phase retrieval methods   

The first method used for phase retrieval is based on an algorithm that is a mixture of the classical Gerchberg–Saxton algorithm (Gerchberg & Saxton, 1972 ▶) and a hybrid input–output algorithm (HIO; Fienup, 1982 ▶), typically denoted as ‘mHIO’ (modified HIO). It has been proven very useful for imaging of microorganisms such as bacteria (Bartels *et al.*, 2012 ▶; Giewekemeyer *et al.*, 2011 ▶). Let 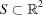 be the support of an isolated specimen, 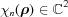 be the current estimate of the object in the object plane and 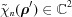 be the current estimate that was propagated (in the Fresnel regime) to the detection plane: 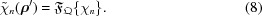 Because of noise in the data, it is convenient to carry out a soft projection onto the measured data 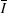 (Giewekemeyer *et al.*, 2010 ▶): 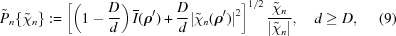
where *D* is a noise-dependent relaxation parameter. The noise parameter determines the influence of the measured data on the current estimate by comparing it to the reconstruction error *d*:
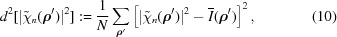
where *N* is the number of measured intensity values and the summation is carried out over all *N* pixels at positions 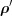. Note that as long as 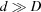 the algorithm ‘prefers’ the measured data. However, when the error decreases to a level where noise in the data becomes important, the weight of the measured data decreases. The complete modulus operator is conveniently written in the plane of the sample as 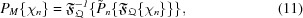
where 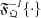 denotes the back-propagation from the detection to the sample plane.

Convergence to a solution, however, necessitates one or more constraints in the plane of the sample. The attenuation length of cellular material such as proteins is comparably large. Hence, attenuation of the X-ray beam may be negligible for bacterial cells with a size in the range of a few micrometres, which can be enforced by the following operator: 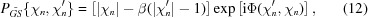
where 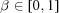 is a feedback parameter, 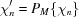 and the phase 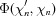 is obtained as described below. The amplitude is thus slowly pushed towards unity with every iteration step. In addition, a support is used on the phase: 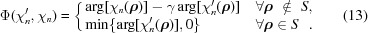
The phase outside the support is pushed to zero with a strength that is determined from the value of the feedback parameter 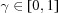. Additionally, the phase is enforced to become negative within the region of the support. Implicitly, it is assumed that the maximum relative phase shift remains below π. Now, a full iteration cycle, 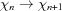, can be written as 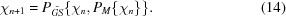
The algorithm is stopped as soon as 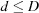.

The second phase reconstruction method that has been applied here is known as ‘holotomography’ (Cloetens *et al.*, 1999 ▶, 2006 ▶; Cloetens, 1999 ▶; Zabler *et al.*, 2005 ▶). The name arises from the applied imaging regime, namely the holographic regime, and from its typical combination with tomography. The underlying phase reconstruction scheme is based on reconstructing two-dimensional projection data and does not necessarily involve an extension to three-dimensional data sets. The phase reconstruction is based on the contrast transfer function of a pure phase object with slowly varying phase (Turner *et al.*, 2004 ▶). In this case, the Fourier transform of the measured intensity in the plane *z* can be approximated by (here the one-dimensional case is given for simplicity) (Cloetens *et al.*, 1999 ▶; Salditt *et al.*, 2009 ▶) 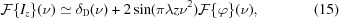
where 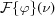 is the Fourier transform of the phase of the object 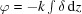, δ is the refractive index decrement and 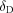 denotes the Dirac delta function. The sinusoidal term has a significant influence on the transfer of the phase of the object in terms of spatial frequencies. For instance, phase contrast of the object is strongly suppressed at the zeros 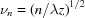, 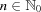. The dependence of the contrast on the propagation distance suggests the need to include measurements from multiple distances 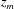. The Fourier representation of the phase function can be obtained by minimizing the following cost function (Zabler *et al.*, 2005 ▶): 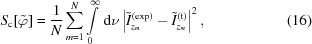
where the tilde denotes the Fourier transform and the summation is over *N* distances 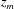. 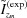 and 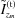 denote the experimental data and the theoretical model according to equation (15), respectively. Minimizing the cost function with respect to 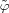, *i.e.* requiring 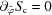, yields the Fourier transform of the phase function (Zabler *et al.*, 2005 ▶): 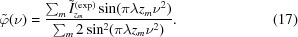
The phase ϕ is thus obtained by an inverse Fourier transform of the right-hand side. In practice, this involves a regularization parameter in the denominator that is frequency dependent and also determined by the experiment.

### 2.4. Sample preparation   

The *B. subtilis* (DSM No. 23778) and *B. thuringiensis* (DSM No. 350) samples were cultivated as follows. Cell material and endospores were harvested after 6 d of aerobic incubation in a rotary shaker at 303 K (200 r min^−1^) in liquid lysogeny broth medium (Harwood & Cutting, 1990 ▶). Excess medium was removed by centrifugation at 3000*g* for 10 min at 277 K. Thereafter, the pellets were washed two times with pure water (centrifugation at 5000*g* for 10 min). In the case of *B. subtilis* envelopes (cells), a layer consisting almost solely of endospores could be extracted after the second centrifugation step. It appears as a dark band on top of the pellet of vegetative cells. The sample material was then stored at low temperature (277–281 K) until further use. Part of the material was put aside for preparation of TEM samples.

**Figure 3:**
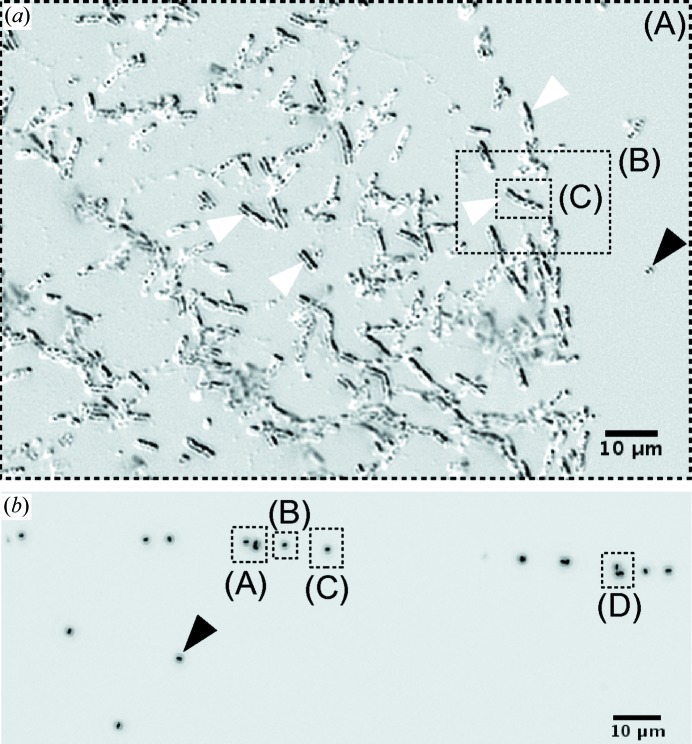
Optical light micrographs (differential interference contrast, 40×) of the samples of (*a*) freeze-dried *B. thuringiensis* cells and endospores and (*b*) freeze-dried *B. subtilis* endospores. The regions that were analysed with waveguide-based imaging are indicated by dashed black frames and labels (A), (B), (C) and (D) in both images. The bacteria and remnant cell material in (*a*) are mainly rod shaped (white arrow heads). Smaller spots are likely to be isolated endospores (black arrow head). An isolated endospore is indicated in (*b*) by a black arrow head.

The samples for X-ray imaging were prepared by suspending about 10–20 µl of the pellet in 100 µl of pure water (for an optimum material density on the sample holder). Next, the samples were plunge frozen using a vitrobot (Leica EM GP, Leica Microsystems). Droplets of 1 µl of cell/endospore suspension were placed on 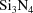 membranes (Silson, UK; membrane thickness 1 µm) inside a humidity- and temperature-controlled chamber (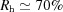, 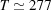
 K) of the vitrobot. After blotting excess liquid, the samples were plunged into a cryogen of 63% propane, 37% ethane (*T* = 80 K) (Tivol *et al.*, 2008 ▶). Samples were freeze dried in a workshop-built freeze drying apparatus. A microscopic inspection of the sample material used for plunge freezing was carried out after freeze drying (Fig. 3 ▶).

### 2.5. TEM imaging   

**Figure 4:**
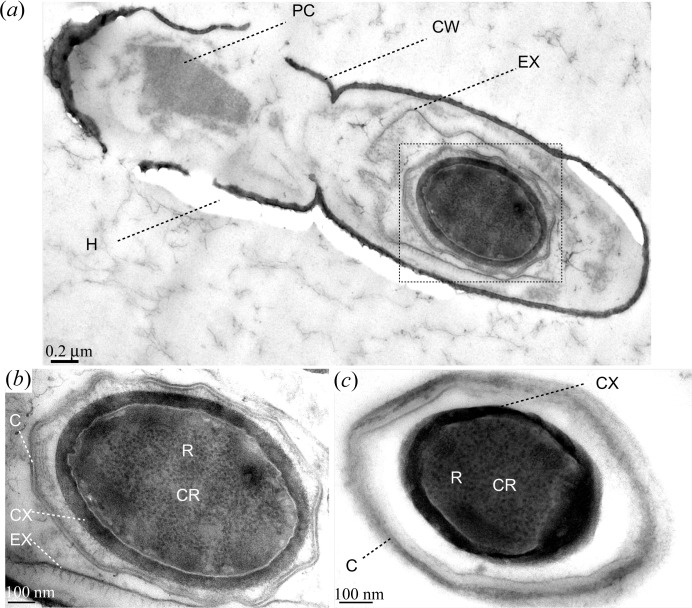
(*a*) A TEM image of two lysing *B. thuringiensis* cells. The cell walls (CW) are already fragmented. One cell contains the typical protein crystal (PC) of *B. thuringiensis* cells, whereas the other cell is occupied by an endospore (dashed frame) that is surrounded by the exosporium (EX). Holes (H) in the TEM slice are visible. The protein crystal has dimensions of about 880 and 440 nm along the major and minor axes, respectively. The extent of the endospore including the coat is about 1190 nm along the major axis and 760 nm along the minor axis. (*b*) A close-up of the endospore as indicated by a dashed frame in (*a*), revealing details of the exosporium (EX), coat (C), cortex (CX) and core (CR). This section of the core contains mainly ribosomes (R). (*c*) A TEM image of an endospore of *B. subtilis*. Structural details including the cortex (CX) and the core (CR) with ribosomes (R) are labelled. The major and minor axes of the core including cortex extend to about 630 and 460 nm, respectively. Taking also the coat into account yields about 1030 nm along the major axis and approximately 700 nm along the minor axis. Scale bars denote 0.2 and 0.1 µm in (*a*) and in (*b*), (*c*), respectively.

The preparation of ultra-thin sections (thickness ∼80 nm) of resin-embedded, chemically fixed and stained samples is a standard procedure for electron microscopy (Hoppert & Holzenburg, 1998 ▶; Kay, 1967 ▶). At first, the bacterial cell material was chemically fixed with 2.5%(*v*/*v*) glutaraldehyde. After an incubation time of 90 min on ice, the material was washed by centrifugation at least once. Next, the excess liquid was almost entirely removed. The remnant pellet was resuspended, and the cell material was then mixed with an equal amount of liquid 2%(*w*/*v*) Bacto-agar (Agar Bacteriological, Oxoid LP0011, UK). The agar was kept in a liquid state at 323 K before being mixed with the cell material. Thereafter, the liquid was allowed to cool and solidify. In the following step, the solidified agar/cell suspension was cut with a razor blade into cubes with side length 1 mm. Next, the samples were dehydrated *via* an ascending series of ethanol concentration: incubation in 15%(*v*/*v*) for 15 min at 273K, 30% for 30 min at 273 K, 50% for 30 min at 253 K, 70% for 30 min at 253 K, 95% for 30 min at 253 K, 97% for 15 min at 253 K, 99% for 15 min at 253 K and 100% for 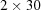
 min at 253 K. Thereafter, dilutions of resin LR White (Agar Scientific, UK) in ethanol followed: 66.6%(*v*/*v*) and 100%, both were incubated for 2 h at room temperature. Single aliquots were then transferred into resin-filled gelantine capsules. Finally, polymerization was carried out by baking the capsules for at least 24 h at 323 K. Cutting of ultra-thin sections from the hardened resin cell block, which was pre-shaped by milling, was carried out with a microtome (Ultracut E, Reichert-Jung). The ultra-thin sections were transferred onto TEM sample grids covered with a thin support film of formvar. Before TEM imaging, the sample sections were negatively stained by incubating them for about 2 min on a droplet of 4%(*w*/*v*) aqueous solution of uranyl acetate. The TEM images (JEM-1011; JEOL, Japan) are presented in Fig. 4 ▶.

### 2.6. Data recording and processing   

In the following, the recorded data are listed. Single diffraction patterns and a series of diffraction patterns with a corresponding series of empty beam intensities were recorded at different source-to-sample distances 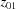 (equivalent to a variation of the magnification) on different positions of both *Bacillus* spp. samples. The experimental parameters are summarized in Tables 1 ▶ and 2 ▶. In addition, the diffraction data of the *B. thuringiensis* sample includes a transverse variation of the sample position. On region (A), 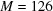, the sample was illuminated at four different overlapping regions by shifting the sample three times by about 150 µm in the horizontal direction.

Next, details about the iterative phase reconstruction and the determination of the support that was necessary for the mHIO algorithm are described. In the case of the *B. thuringiensis* data, a semi-automatic procedure was applied to obtain an accurate support function. Let 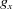, 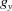 be the gradient of the holographically reconstructed phase. Then it was found that the ‘radial’ gradient 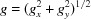 information can be used to locate the cellular structures in the holographic reconstructions as exemplified in Figs. 2 ▶(*b*) and 2 ▶(*c*). A first support estimate was obtained by creating a binary mask from *g* by setting a threshold, followed by a dilation of the binary data and, finally, filtering using MATLAB (The MathWorks Inc., Natick, MA, USA) routines. The final support was obtained similarly by using a preliminary iterative reconstruction with the first support guess (Fig. 2 ▶ *d*). In the case of the *B. subtilis* data, the gradient method was not found to be practical because of the low gradient values of the sample in comparison to the holographic background. Instead, a simple threshold for the holographically reconstructed phase was set, followed by dilation and filtering of the binary mask. The reconstructions were carried out at a maximum of 2000 iterations using the parameters 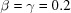. The parameter *D* of the soft projection onto the modulus of the measured diffraction data was set manually. Here it should be noted that the algorithm clearly diverges within a few hundred iterations if *D* is chosen too small. The optimum *D* is thus found by taking a slightly larger value above the threshold where divergence is observed. At the last iteration, error values in the range of 0.015–0.15 were obtained. An mHIO reconstruction with 2000 iterations of a hologram of 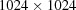 pixels takes about 24 s using GPU computing on an NVDIA Geforce GTX TITAN Black with 6 GB of memory. Prior to merging the *B. thuringiensis* data that were taken at four different positions on region (A) at constant 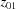, the displacement vectors between the neighbouring phase reconstructions were calculated by using the method of discrete Fourier transform registration (Guizar-Sicairos *et al.*, 2008 ▶).

### 2.7. Determination of dose and mass densities   

Next, the determination of the applied dose during the measurements is discussed. The photon flux was measured with the Pilatus 300K during both experiments. The photon fluxes corresponding to the smaller field of view of the Photonic Science detector were calculated as 1.43 × 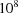
 photons s^−1^ and 2 × 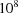
 photons s^−1^ in the case of the *B. thuringiensis* and the *B. subtilis* data, respectively. Doses were then determined according to the following equation by assuming the photon flux to be constant over the field of view and taking the tabulated absorption coefficient for the model protein 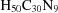 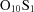 of mass density 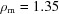
 g cm^−3^ (Howells *et al.*, 2009 ▶): 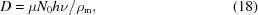
where μ is the absorption coefficient, 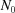 is the number of incident photons per unit area and 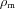 is the mass density of the sample. Results are listed in Tables 1 ▶ and 2 ▶.

Calculation of the projected two-dimensional mass densities 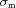 from the reconstructed phase maps was performed as outlined by, for example, Giewekemeyer *et al.* (2010 ▶): 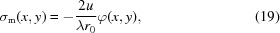
where 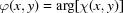 is the reconstructed two-dimensional phase of the object function 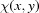, and 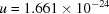
 g, 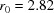
 fm and 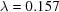
 nm denote the atomic mass unit, the classical electron radius and the wavelength, respectively. A 10% error according to Giewekemeyer *et al.* (2010 ▶) was assumed for the integral mass values (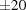
 fg).

## 3. Results   

**Figure 5:**
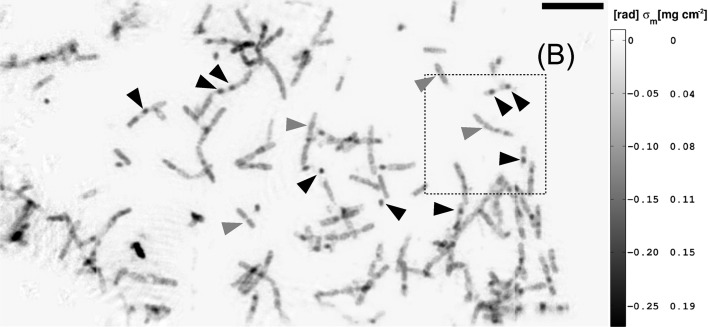
Region (A): the image shows a phase map of the *B. thuringiensis* sample. The phase map corresponds to four single mHIO reconstructions that have been merged in the same plane. Grey arrow heads point to some of the rod-shaped remnant cell material. The black arrow heads indicate electron-dense cellular features such as endospores. The dashed black frame indicates region (B), including (C), of the sample (*cf.* Fig. 7 ▶). The scale bar denotes 10 µm.

**Figure 6:**
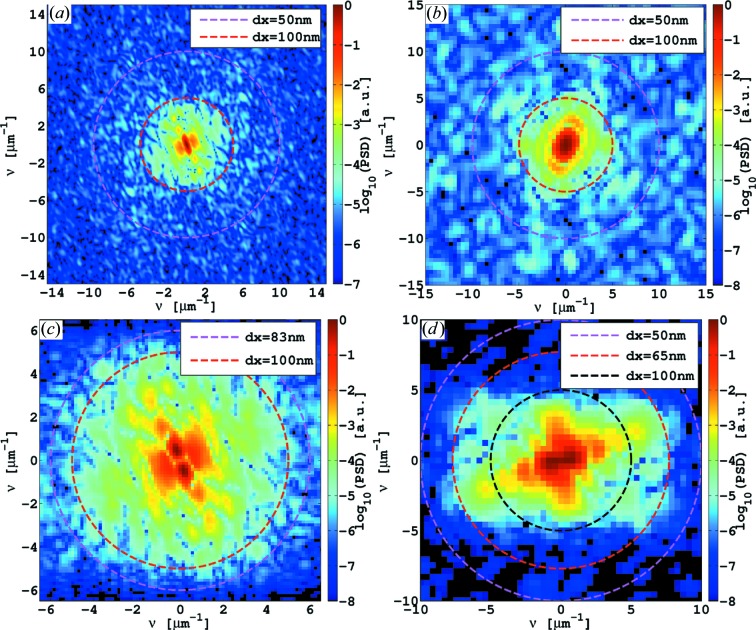
(*a*) and (*b*) PSDs of mHIO reconstructions of *B. thuringiensis* [region (C), Fig. 7 ▶(*b*)] and *B. subtilis* data [region (B), Fig. 8 ▶(*b*)], indicating resolutions in the range of 100 nm (half-period). (*c*) and (*d*) PSDs of CTF-based reconstructions of the *B. thuringiensis* data [region (C), Fig. 9 ▶(*b*)] and *B. subtilis* data [region (A), Fig. 10 ▶], indicating resolutions of about 100 and 60 nm (half-period), respectively.

At first, the results of the mHIO reconstructions of the *B. thuringiensis* data are addressed. Three regions, (A), (B) and (C) [*cf.* the optical light micrographs of Fig. 3 ▶(*a*)], on the sample of the unstained, unsliced and freeze-dried bacterial material were successfully reconstructed. Fig. 5 ▶ shows the phase map corresponding to four single mHIO reconstructions. Together, the full field of view covers about 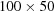
 µm at a magnification of 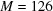 (Table 1 ▶). Resolution estimates from the power spectral density (PSD) of the data are exemplified in Fig. 6 ▶ (Giewekemeyer, 2011 ▶; Wilke *et al.*, 2012 ▶). Analysing a large part of the phase map (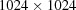 pixels) indicates that most of the frequency contributions are contained inside a ring of 238 nm half-period. The sample structure correlates very well with the optical light micrographs. For instance, most of the rod-like cell remnants within region (B) can be identified both in Fig. 3 ▶(*a*) and in Fig. 5 ▶. In particular, this is the case for region (C) within (B). In general, many remnants of the rod-like bacterial cells can be identified with varying contrast in the reconstruction. Single cells abundantly appear to be connected in strands of two or more cells. Because of the prepared state of the cell culture (late stationary phase), many of the cells may already be lysing or empty. The calculated projected mass density (phase shift) shows cells of different density or multiple cells lying on top of each other. In addition, some smaller spots within the cells exhibit a comparably high mass density (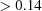
 mg cm^−2^). On this sample the high-mass-density objects are supposedly endospores or, depending on the size, possibly protein crystals, as will become clear after discussing the phase maps with higher resolution.

**Figure 7:**
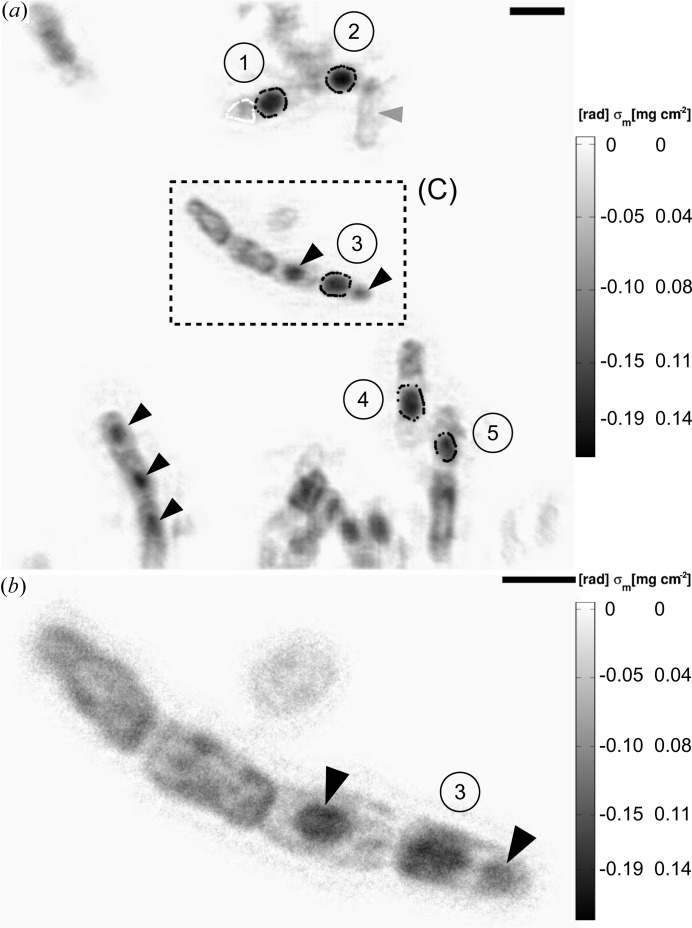
(*a*) Region (B): the figure shows a phase image of the *B. thuringiensis* sample. The phase map corresponds to a single mHIO reconstruction. The black dots indicate the boundaries of the endospores used for mass estimations whereas the white dots surround the region being defined here as ‘background’ (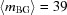
 fg). Endospores are labelled from (1) to (5). The average mass per single endospore is 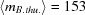
 (9) fg. The black arrow heads indicate positions of other dense bacterial features such as possible BT crystals. The grey arrow head points to an almost fully lysed cell. The scale bar denotes 2 µm. (*b*) Region (C) of (*a*): the figure shows a phase image of the *B. thuringiensis* sample. The phase map corresponds to a single mHIO reconstruction at higher magnification. The scale bar denotes 1 µm.

The mass density map of region (B), which was taken at a magnification of 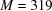, is presented in Fig. 7 ▶(*a*). Its power spectral density indicates that the overall resolution is about 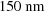. On the mass density map, five ellipsoid structures were attributed to intracellular endospores [labelled as (1)–(5)]. The mass of these endospores was determined by integrating the projected mass density map as indicated by black dots surrounding the endospores. Material of the cell walls below and on top of the endospores was accounted for by subtracting a background of 39 fg, which was obtained from the same map. As a result, the dry masses of individual endospores were obtained in the range of ∼110–180 (20) fg with an average mass of 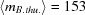
 (9) fg for a single, dry endospore (Table 3 ▶). In addition to endospores, other smaller dense features are visible on the mass density map, as indicated by black arrow heads in Fig. 7 ▶(*a*). These features may well be indicative of the BT crystals formed by *B. thuringiensis*, as can be observed in a TEM image (Fig. 4 ▶ *a*).

Another reconstructed mass density map taken on region (C) at a magnification of 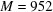 is shown in Fig. 7 ▶(*b*). Its power spectral density (Fig. 6 ▶ *a*) indicates a resolution down to 100 nm (half-period) and slightly better. In comparison to the preceding reconstruction of Fig. 7 ▶(*a*), the resolution is clearly improved. A strand of four partly lysed bacterial cells can be identified. Two of these cells include dense features (*cf.* black arrow heads) like the one that is labelled as the endospore (3). These may be attributed to the BT crystals.

**Figure 8:**
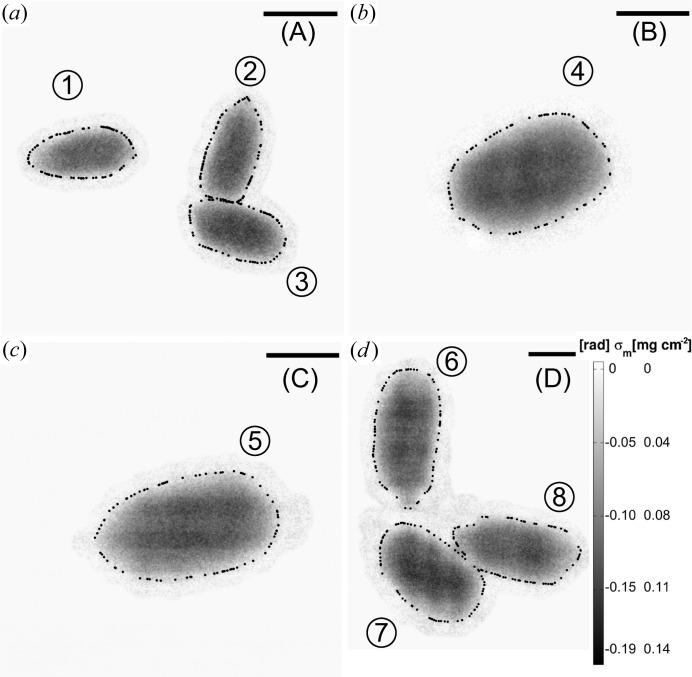
The figure presents mHIO phase reconstructions of endospores of *B. subtilis* of regions (A), (B), (C), (D) (*cf.* Fig. 3 ▶ *b*). The endospores are labelled from (1) to (8). The black dots indicate the boundaries used for mass estimations. The average mass per single endospore is 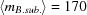
 (7) fg. The colour bar is the same in (*a*), (*b*) and (*c*) as in (*d*). The scale bars in (*a*), (*b*), (*c*) and (*d*) denote 1, 0.5, 0.5 and 0.5 µm, respectively.

Next, the results of the mHIO reconstructions of the *B. subtilis* data from regions (A), (B), (C) and (D) are given. The optical light micrograph of Fig. 3 ▶(*b*) clearly shows isolated endospores on all these regions. The phase reconstructions are summarized in Fig. 8 ▶. In total, eight individual endospores can clearly be identified [labels (1)–(8)]. Their projected mass density extends up to 0.19 mg cm^−2^. The endospores appear without the cell material of the mother cell as expected. For this reason, the endospore masses were obtained without background subtraction. Mass results of the dried endospores in the range of ∼150–190 (20) fg are listed in Table 4 ▶. An average mass of 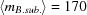
 (7) fg per single dried endospore was obtained. Notably, the endospores differ slightly in shape and size. A determination of the resolution by PSDs as presented in Fig. 6 ▶(*b*) reveals frequency contributions down to 100 nm (half-period). The internal structure of the endospores cannot clearly be identified. However, the inner parts of the endospores appear slightly denser after a small transition zone, which may indicate the transition from coat to cortex and the centre of the endospore.

**Figure 9:**
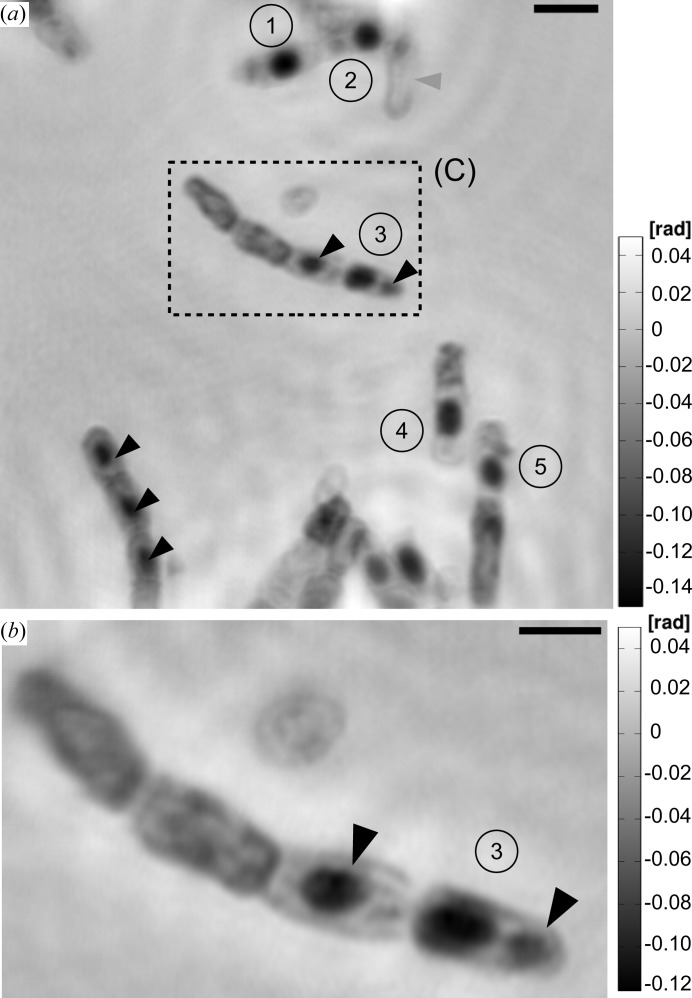
(*a*) Region (B): the image shows a phase reconstruction using the CTF-based reconstruction of the *B. thuringiensis* sample. The scale bar denotes 2 µm. (*b*) Region (C) of (*a*) but at higher magnification. Note that the density of the core of the endospore (3) appears slightly higher than the outer part. The electron-dense feature (black arrow head) on the right-hand side of the spore (3) is probably a BT crystal, as the cell already includes one endospore. The scale bar denotes 1 µm. In both images the labelling is the same as in Fig. 7 ▶.

**Figure 10:**
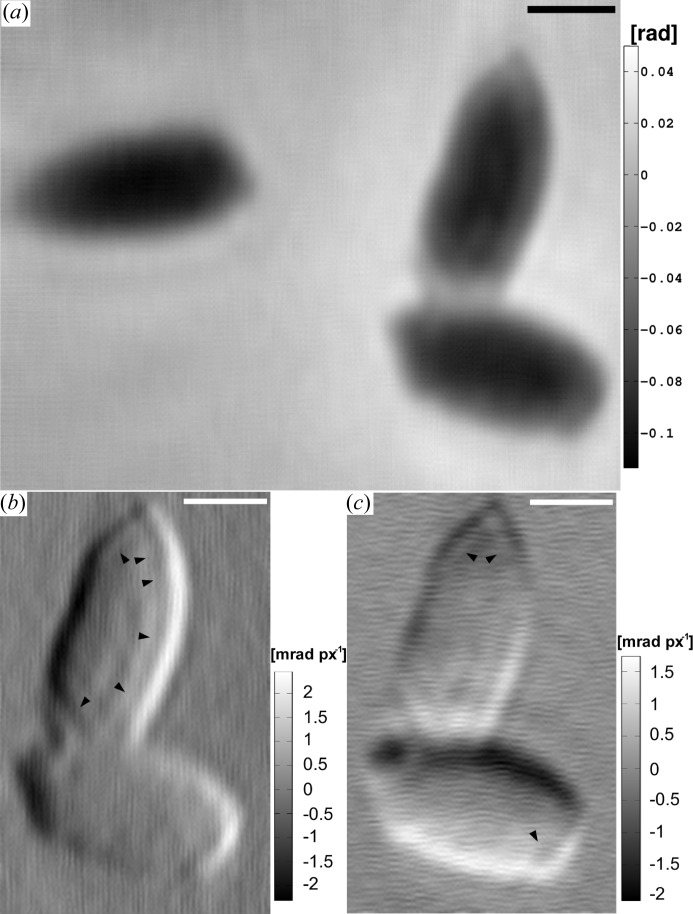
(*a*) The CTF-based reconstruction of region (A) of the *B. subtilis* sample. Three isolated endospores can be seen. (*b*), (*c*) Calculated gradients (filtered with 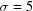
 pixel Gaussian) in the horizontal and vertical directions of the region of two endospores of (*a*). The black arrow heads highlight the transition between two different structural regions of the endospore that are attributed to the coat and the inner part. The repetitive structure on the gradient maps is an artefact of the reconstruction in (*a*). The scale bars denotes 0.5 µm.

Finally, the phase reconstructions using the CTF-based method follow. Figs. 9 ▶(*a*) and 9 ▶(*b*) show reconstructions of the *B. thuringiensis* sample at final magnifications 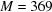 and 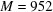 using four and two different planes 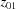, respectively. The visual impression of the images is quite good in comparison to the mHIO reconstructions of Figs. 7 ▶(*a*) and 7 ▶(*b*). In particular, the noise in the data is significantly reduced, which makes it easier for the eye to identify the cells and intracellular components such as endospores. However, the overall phase shift deviates slightly from the preceding iterative mHIO reconstructions due to a possibly non-optimum choice of regularization parameters. An inspection of the PSDs suggests an overall achieved resolution of about 135 nm for the data of Fig. 9 ▶(*a*) and of about 100 nm for the data of Fig. 9 ▶(*b*) (*cf.* Fig. 6 ▶ *c*). In comparison to the mHIO reconstructions, the slightly improved resolution is in agreement with the applied doses of about 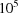
 Gy, which are by about one order of magnitude higher for the case of the reconstruction scheme based on the CTF (*cf.* Table 1 ▶). The reconstruction of Fig. 9 ▶(*b*) may still be improved as here only data sets from two planes (instead of four) were available. The reconstruction of region (A) of the *B. subtilis* sample of dried endospores is presented in Fig. 10 ▶(*a*) (final magnification 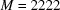). The image shows the three endospores of the mHIO reconstruction in Fig. 8 ▶(*a*) with a clearly reduced noise level. Here, the PSD of the region with the two endospores (2) and (3) (Fig. 6 ▶ *d*) indicates a resolution down to about 65 nm (half-period), which is in good agreement with the improved visual impression. The contrast of the phase map clearly allows us to identify two regions within the spore (2) that may be linked to the coat and the interior, namely the cortex and the core. These two structural regions of the endospore can be better distinguished in Figs. 10 ▶(*b*) and 10 ▶(*c*), which show the horizontal and vertical gradients (filtered with 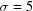
 pixel Gaussian). Size measurements from the reconstruction of Fig. 10 ▶(*a*) yield about 270 nm for the interior and 170–200 nm for the outer part (coat) along the horizontal direction.

## 4. Conclusion, discussion and outlook   

In conclusion, two different waveguide-based X-ray imaging approaches have been successfully demonstrated on two unstained, unsliced and freeze-dried samples of different endospore-forming bacterial species, namely *B. thuringiensis* and *B. subtilis*. The versatility of the waveguide imaging scheme in terms of magnification allowed for obtaining either a large field of view for an inspection of many bacterial cells at the same time or a small field of view that can be used for imaging single cells at high resolution. A maximum resolution was achieved by using the CTF-based reconstruction, yielding about 65 nm (half-period) on single endospores of *B. subtilis*. In this case, structural changes from what we supposed to be coat to cortex and core began to be observable. The applied dose of about 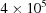
 Gy is an order of magnitude lower than the value of 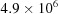
 Gy which we reported for ∼50 nm resolution on freeze-dried bacteria *Deinococcus radiodurans* (Wilke *et al.*, 2012 ▶) using the ptychographic method for phasing farfield diffraction data. In the case of the *B. thuringiensis* data, the achieved resolution was lower but sufficient to distinguish different cellular components such as endospores. Indications of other possible intracellular occurrences such as BT crystals were also found, but further validation is needed, by either X-ray imaging data at higher resolution, a combination with tomography or nano-diffraction.

In addition, the iterative phase reconstructions using the mHIO algorithm could be used to determine the masses of entire individual endospores of both bacterial species. Values in the range of ∼110–190 (20) fg were obtained, yielding average values for single endospores of 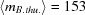
 (9) fg and 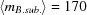
 (7) fg. These values can be compared with measurements of the wet weight and wet and dry density of endospores (Carrera *et al.*, 2008 ▶; Tisa *et al.*, 1982 ▶). Carrera *et al.* (2008 ▶) used Percoll gradients to determine the mass density of comparably large numbers of endospores from *B. thuringiensis* (4055) and *B. subtilis* (1031). They calculated the mass of a single endospore from the determined density by using an ellipsoid size model with size estimations from electron microscopy studies on chemically fixed and air-dried samples (Carrera *et al.*, 2007 ▶). They determined wet weights of 643 fg (wet density 1.17 g ml^−1^) and 196 fg (wet density 1.22 g ml^−1^, dry density 1.52 g ml^−1^) for *B. thuringiensis* and *B. subtilis*, respectively. A dry density of *B. thuringiensis* endospores was not obtained (Carrera *et al.*, 2008 ▶; Tisa *et al.*, 1982 ▶). Therefore, a direct comparison cannot be made for *B. thuringiensis* endospores, but it is noted that there is a relatively large gap between the wet weight of 643 fg and the determined dry weight value of 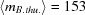
 (9) fg. The mass difference of 490 fg would correspond to a volume of about 0.49 µm^3^ of water (*V* [µm^3^] = 10^−3^ *m* [fg]/ρ [g cm^−3^]). However, one obtains a similar volume here if one applies the ellipsoid model 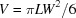 (*L* major and *W* minor axis) of Carrera *et al.* (2008 ▶) to the projections of the dried endospores. That is, the mass difference could be explained by the difference between the dried and wet state of the endospore, but it would require the endospores to be filled with a comparably large amount of water. In the case of the *B. subtilis* endospores, the comparison with the data from Carrera and co-workers is equally surprising. For instance, the volume estimations that were used by Carrera *et al.* for the determination of the wet weight would yield a dry weight of 243 fg for endospores of *B. subtilis* of dry density 1.52 g ml^−1^, which is close to the value found here. Taking also into account the reduced size of a dried endospore (in comparison to its wet state) would indicate that the dry weight must be even lower than 243 fg. However, the ellipsoid model can also be used to determine the dry density of our data. This yields for the endospore (2) with 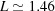
 µm and 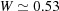
 µm a dry density of ρ [g ml^−1^] = 10^−3^ *m* [fg]/*V* [µm^3^] = 180/0.214 = 0.84, which is significantly below the average spore density of 1.52 g ml^−1^ (Carrera *et al.*, 2008 ▶). Hence, the conclusion is that in both cases the mass estimations appear to be surprisingly low, but there are also other factors that influence the weight of the endospores. For instance, the differences probably arise from the comparison of endospores from two different *B. subtilis* strains. Moreover, it is known that the used sporulating medium has an influence on, for example, the coat of the endospore (Carrera *et al.*, 2008 ▶; Driks, 1999 ▶; and references therein). Here, the preparation of the endospores differs from that described by Carrera *et al.* (2007 ▶, 2008 ▶) by working without a special sporulating medium (Driks, 1999 ▶) and by using a different growth medium. Differences in the size and density of the endospores are thus expectable. As indicated above, the water content of the endospores affects the wet weight and the wet density, too. In addition, it is known that the endospore volume depends on its hydration level. According to Plomp *et al.* (2005*b* ▶) and references therein, endospores undergo swelling when exposed to water. Consequently, some volume may be filled with water and the weight thus increases. Furthermore, we demonstrated accurate density estimations within a range of 10% of the theoretical expectation as determined from measurements on an Au test sample (Bartels, 2013 ▶; Bartels *et al.*, 2015 ▶), which underpins the accuracy of the applied phasing scheme. In principle, the CTF-based reconstructed phase maps are also quantitative (Cloetens *et al.*, 1999 ▶, 2006 ▶), but because of a possibly non-optimum choice of regularization parameters, small differences in comparison to the mHIO phase reconstructions were obtained. For this reason, the phase maps have not been converted to projected mass density maps yet.

In the future, imaging of hydrated specimens is an appealing prospect. In view of the current resolution by TEM imaging, improvements still need to be made. Increasing the resolution and a combination with tomography may require preparation of frozen hydrated samples but could allow for determination of the water content of the endospores and their core, which is not easily accessible otherwise, for instance, by TEM imaging. Moreover, a combination with cellular nanodiffraction may allow for a clear identification of BT crystals.

## Figures and Tables

**Table 1:** Experimental parameters of the *B. thuringiensis* data 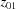
 is the distance between the source and the sample. The distance 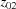 between the sample and the detector was in all cases 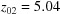
m. *M* is the magnification. *N* is the number of images that were recorded. The integral doses of the reconstructions of Figs. 5 ▶, 9 ▶(*a*) and 9 ▶(*b*) are of the order of 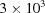 (mean overlap of two adjacent reconstructions), 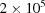 and 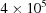
Gy, respectively.

Region	Figure	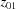 (mm)	*M*	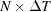 (s)	Fluence (photonsnm 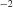 )	Dose (Gy)
(A)	5 ▶	40	126	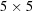	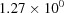	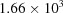
(A)		40	126	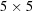	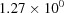	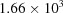
(A)		40	126	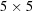	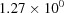	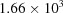
(A)		40	126	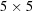	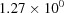	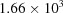

(B)	7 ▶(a)	15.80	319	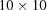	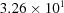	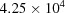
(C)	7 ▶(*b*)	5.30	952	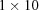	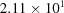	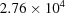

(B)	9 ▶(*a*)	13.70	369	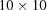	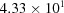	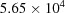
(B)		13.80	366	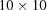	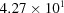	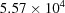
(B)		14.80	341	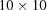	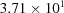	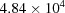
(B)		15.80	319	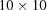	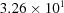	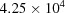

(C)	9 ▶(*b*)	5.30	952	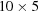	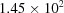	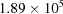
(C)		5.40	935	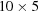	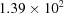	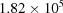

**Table 2:** Experimental parameters of the *B. subtilis* data 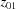
 is the distance between the source and the sample. The distance 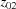 between the waveguide and the detector was in all cases 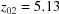
m. *M* is the achieved magnification. *N* is the number of images that were recorded. The integral dose for the CTF-based reconstruction (Fig. 10 ▶) is of the order of 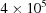
Gy.

Region	Figure	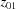 (mm)	*M*	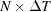 (s)	Fluence (photons nm 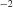 )	Dose (Gy)
(A)	8 ▶(*a*)	3.56	1442	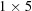	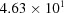	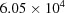
(B)	8 ▶(*b*)	1.81	2836	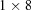	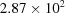	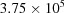
(C)	8 ▶(*c*)	1.81	2836	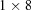	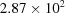	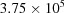
(D)	8 ▶(*d*)	2.56	2005	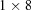	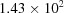	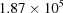

(A)	10 ▶	2.31	2222	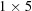	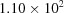	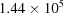
(A)		2.56	2005	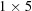	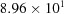	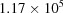
(A)		2.81	1827	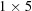	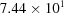	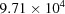
(A)		3.06	1677	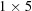	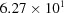	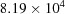

**Table 3:** Results of mass measurements of individual endospores of *B. thuringiensis* The average mass per single endospore is 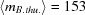
(9)fg.

Region	Figure	Label	Mass (fg)
(B)	7 ▶(*a*)	(1)	181 (20)
		(2)	166 (20)
		(3)	130 (20)
		(4)	174 (20)
		(5)	113 (20)

**Table 4:** Results of mass measurements of individual endospores of *B. subtilis* The average mass per single endospore is 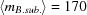
(7)fg.

Region	Figure	Label	Mass (fg)
(A)	8(*a*)	(1)	153 (20)
		(2)	180 (20)
		(3)	192 (20)

(B)	8(*b*)	(4)	158 (20)

(C)	8(*c*)	(5)	154 (20)

(D)	8(*d*)	(6)	185 (20)
		(7)	189 (20)
		(8)	153 (20)
